# The origin recognition complex in human diseases

**DOI:** 10.1042/BSR20130036

**Published:** 2013-06-11

**Authors:** Zhen Shen

**Affiliations:** Laboratory of Biochemistry and Molecular Biology, The Rockefeller University, New York, NY 10065, U.S.A.

**Keywords:** African trypanosomiasis, American trypanosomiasis, DNA replication, Epstein–Barr virus, Meier–Gorlin syndrome, origin recognition complex, pre-replication complex, AML, acute myeloid leukaemia, ARS, autonomously replicating sequence, BAH, bromo adjacent homology, Cdc6, cell division cycle 6, CdLS, Cornelia de Lange syndrome, CDK2, cyclin-dependent kinase 2, CID, CDK inhibitory domain, Cdt1, chromatin licensing and DNA replication factor 1, DS, dyad symmetry, EBV, Epstein–Barr virus, EBNA1, Epstein–Barr nuclear antigen 1, H4K20me2, H4K20 di-methylation, KSHV, Kaposi’s sarcoma-associated herpesvirus, LANA, latency-associated nuclear antigen, LRWD1, leucine-rich repeats and WD-repeat-domain-containing 1, MDS, myelodysplastic syndrome, MCM, minichromosome maintenance, MGS, Meier–Gorlin syndrome, NIPBL, nipped-B-like, ORC, origin recognition complex, PARP, poly(ADP-ribose) polymerase, pre-RC, pre-replication complex, RG-rich motifs, arginine- and glycine-rich motifs, RNAi, RNA interference, SNP, single nucleotide polymorphism, TRF2, telomere repeat factor 2, VSG, variant surface glycoprotein

## Abstract

ORC (origin recognition complex) serves as the initiator for the assembly of the pre-RC (pre-replication complex) and the subsequent DNA replication. Together with many of its non-replication functions, ORC is a pivotal regulator of various cellular processes. Notably, a number of reports connect ORC to numerous human diseases, including MGS (Meier–Gorlin syndrome), EBV (Epstein–Barr virus)-infected diseases, American trypanosomiasis and African trypanosomiasis. However, much of the underlying molecular mechanism remains unclear. In those genetic diseases, mutations in ORC alter its function and lead to the dysregulated phenotypes; whereas in some pathogen-induced symptoms, host ORC and archaeal-like ORC are exploited by these organisms to maintain their own genomes. In this review, I provide detailed examples of ORC-related human diseases, and summarize the current findings on how ORC is involved and/or dysregulated. I further discuss how these discoveries can be generalized as model systems, which can then be applied to elucidating other related diseases and revealing potential targets for developing effective therapies.

## INTRODUCTION

DNA replication in eukaryotic organisms begins with the loading of the pre-RC (pre-replication complex) at the replication origins. This process requires the ordered assembly of ORC (origin recognition complex), Cdc6 (cell division cycle 6), Cdt1 (chromatin licensing and DNA replication factor 1), MCM2–7 (minichromosome maintenance 2–7) to replication initiation sites in late M to G_1_ phase [1–4].

ORC, a six-subunit complex, functions as the initiator in recognizing replication start sites as well as interacting with subsequent replication factors (pre-RC components) [4]. It was first discovered in the unicellular model organism *Saccharomyces cerevisiae* as a multi-protein complex binding to ARS (autonomously replicating sequence) [5]. Subsequently, most ORC orthologues have been identified in other eukaryotic organisms [6]. Although functionally conserved, the mechanisms of how ORC binds to replication origins are highly diverse. In contrast with ARS in *S. cerevisiae*, the binding mechanism and the consensus origin sequence in higher eukaryotes remains a mystery. In search for the answers, additional factors have been reported over the past few years in order to delineate the molecular mechanism [7], including the ORC-associating protein ORCA/LRWD1 (leucine-rich repeats and WD-repeat-domain-containing 1) that stabilizes ORC binding to chromatin in human cells [8–11].

Interestingly, ORC also exhibits non-replication functions [12], including the involvements in transcription silencing and heterochromatin formation [13–18], chromosome condensation and chromatid cohesion [19–28], centrioles and centrosomes [29,30], telomere function [8,31,32], neuron development [33–36] and cytokinesis [37–41]. Remarkably, some of these functions are independent of ORC's intrinsic replication property and are therefore genetically separable, demonstrating a much more complicated ORC network.

Cdc6 and Cdt1 are important replication licensing factors, with protein levels and cellular localizations fluctuating in a cell-cycle-dependent manner [4]. When deregulated, they can lead to re-replication and genomic instability, linking these factors to various types of human cancers [42]. However, ORC and its relation to human diseases are just beginning to be investigated and understood. In this review, I provide several examples of ORC-related human diseases. Of note, in some cases, ORC mutations are the primary cause; while in other cases, host ORCs are exploited by the pathogens. For each instance, I further discuss how the molecular mechanism can be generalized as the model system, applied to other human diseases and utilized for developing potent therapeutic approaches.

## MEIER–GORLIN SYNDROME (MGS): A LESSON FROM ORC MUTATIONS

MGS, also known as ear-patella-short stature syndrome, was first reported by Meier et al. in 1959 [43] and Gorlin et al. in 1975 [44]. Characterized by bilateral microtia, aplasia/hypoplasia of the patellae and prenatal and postnatal growth retardation [45,46], it is considered a rare autosomal recessive disorder based on its occurrence in siblings with equal sex ratio [47].

Sequencing of the MGS patients identified mutations in *ORC1*, *ORC4*, *ORC6*, *CDC6* and *CDT1* genes [46,48,49]. In contrast with truncation and splicing mutations, site-specific missense mutations that result in amino acid residue substitutions are more dominant in MGS, including E127G and R105Q in Orc1, Y174C in Orc4, Y232S in Orc6, T323R in Cdc6 and R462Q in Cdt1 [48,50,51]. This is consistent with the essential cellular functions of DNA replication machinery, and is further suggestive of functional domains underlying these specific single mutations. Among these five genes, mutations in *ORC1* as well as *ORC4* have been extensively investigated [46].

**Figure 1:**
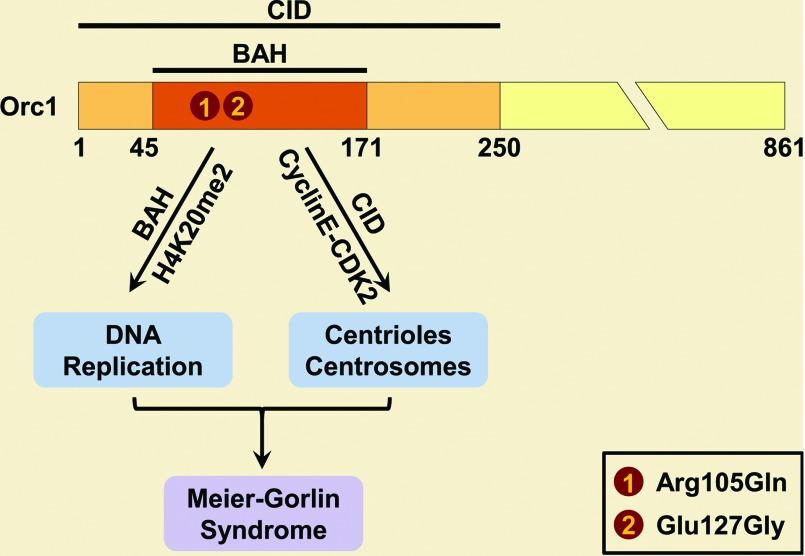
Orc1 mutations in MGS Missense mutations E127G and R105Q occur within the BAH domain as well as the CID. The Orc1 BAH domain binds to H4K20me2, which is important for ORC chromatin association, DNA replication and cell-cycle progression. On the other hand, the R105Q mutation in the CID can specifically abolish Orc1 inhibition of cyclin E-CDK2 kinase, and ectopically expressing this mutant causes reduplication of centrioles/centrosomes and slower cell proliferation. Therefore Orc1 deregulation from both cellular pathways lead to reduced and insufficient cell growth, which could be one major cause of MGS.

Both Orc1 E127G and R105Q mutations exhibit reduced chromatin binding of Orc1, diminished pre-RC assembly and impaired activation of replication origins [50]. Consequently, these Orc1-deficient cells show a slower S-phase progression compared with normal cells [50]. Interestingly, depletion of Orc1 by injecting morpholino oligonucleotides causes dwarfism in zebrafish, similar to the phenotype of MCM5 depletion [50], indicating that these growth defects are the results from (at least partially) the pre-RC pathway. In addition, since both E127G and R105Q mutations take place in the Orc1 N-terminal BAH (bromo adjacent homology) domain, studies concentrated on this domain have unveiled more detailed insights. The Orc1 BAH domain binds to H4K20me2 (H4K20 di-methylation) with high specificity and affinity, as demonstrated from *in vivo* association as well as crystal structural analysis [52]. Investigation using H4K20me2-binding-pocket mutants (Y64A and W88A) reveal that Orc1–H4K20me2 interaction is essential for ORC chromatin association and cell-cycle progression [52]. The functional relevance of this Orc1–H4K20me2 interaction is further tested in zebrafish. When human Orc1 mRNA is co-injected with zebrafish Orc1 morpholino oligonucleotides, the dwarf phenotype can be partially rescued compared with Orc1 morphant alone. However, Orc1–Y64A or Orc1–W88A mRNA is not able to rescue Orc1 morphants, exaggerating the growth defect instead [52]. Together with the fact that ORC subunits associate with histone marks [9–11,53,54], these results suggest that histone modifications may play a direct role in proper DNA replication through the regulation of ORC. Interruptions of these interactions (like Orc1–H4K20me2) that lead to defective cell-cycle progression or cell proliferation may explain the distorted growth in MGS (Figure 1).

Another missense mutation in human Orc4 Y174C was also identified through sequencing, and structural analysis also predicts this mutation to be pathogenic [51]. Orc4 contains a consensus AAA+ domain, which belongs to the AAA+ family (ATPases associated with a variety of cellular activities) that is pivotal to the initiation of eukaryotic DNA replication. The amino acid residue Tyr^174^ is between the Walker B motif and sensor I of the AAA+ domain, which may be responsible for interacting with a conserved arginine residue on an adjacent helix structure [3,4,51,55–57]. To further test this hypothesis, an equivalent missense mutation in *S. cerevisiae* was generated (*orc4*^Y232C^) according to its sequence conservation with higher eukaryotes. Indeed, genetic analyses reveal that this strain undergoes significant defect in G_1_ to S phase progression, and hence a reduced growth rate [51,58]. Therefore the point mutation on this residue is likely to cause the pathological effect.

Additional variants were also reported in Orc6, Cdc6 and Cdt1 [48,49,51]; however, the underlying molecular mechanism is not clear. One possibility, as discussed above, could be the impaired function of the pre-RC that leads to impaired DNA replication and cell-cycle progression. When rapid cell proliferation is on demand, especially at the early stages of development, insufficient growth may result in symptoms seen in MGS patients.

Notably, the most severe growth retardation was observed in individuals with *ORC1* mutations [49], indicating additional dysregulation may exist. In fact, Orc1 controls cyclin E-CDK2 (cyclin-dependent kinase 2)-dependent centriole and centrosome duplication in human cells [29]. The N-terminal 1–250 region of Orc1 is necessary and sufficient to inhibit both cyclin E-CDK2 and cyclin A-CDK2 kinase activities, thus called CID (CDK inhibitory domain) [30]. The R105Q mutation, which lies within the CID, can specifically abolish the Orc1 inhibition of cyclin E-CDK2 kinase but not cyclin A-CDK2 kinase. Ectopically expressing this mutant causes reduplication of centrioles/centrosomes and slower cell proliferation [30]. These data clearly demonstrate that reduplicated centrioles and centrosomes could be another reason for the onset of MGS (Figure 1). Similarly, Orc4 associates with neuronal membranes and is required for proper dendritic growth and branching [36]; whereas Orc6 localizes to kinetochores and is involved in cytokinesis [37–41]. These findings further raise the question of whether these non-replication effects can also contribute to the emergence of MGS.

Another possibility is from the perspective of gene expression. ORC is involved in transcription silencing and heterochromatin formation [13–18], as well as chromosome condensation and chromatid cohesion [19–28]. The study of mutations within the Orc1 BAH domain also indicates the connection between ORC and chromatin modifications [52]. Therefore it is highly possible that mutations in ORC could potentially modulate a subset of gene expression via the alteration of chromatic contexts, which in turn results in the developmental defects seen in MGS. If this is the case, then the aetiology of this syndrome may, to a great extent, be comparable with the role of cohesion proteins in the CdLS (Cornelia de Lange syndrome) and RBS/SC (Roberts-SC phocomelia syndromes) [59–64]. Taking CdLS as an example, it is a dominant genetic disorder characterized by growth and mental retardations among other developmental anomalies [65,66]. Mutations in the cohesin structural components SMC1A, SMC3 and the cohesin regulator NIPBL (nipped-B-like) are the three major mutated proteins causing the CdLS, with the latter leading to the most severe defects [67–70]. Other than its canonical role is regulating sister chromatid cohesion, cohesin also regulates gene expression. For instance, gene expressions are significantly altered in *Drosophila* BG3 cells upon cohesin knockdown [71], and genome-wide analysis in cohesin and NIPBL-mutated human cells also revealed a large number of dysregulated gene expressions [72]. These data indicate that cohesin mutations disturb the expression of critical developmental genes, and hence is a major cause of the CdLS. Similarly, ORC-mutation-mediated alterations of chromatin structure and transcription regulation may also be an underestimated trigger for MGS, and genome-wide gene-expression analysis would be a necessary approach to understanding its molecular basis.

In addition to the mutations of pre-RC components in MGS, several other diseases were linked to variations/mutations of individual ORC subunits. First, a SNP (single nucleotide polymorphism) within the *ORC3* gene has been associated with schizophrenia [73]. The *Drosophila* Orc3 homologue latheo was first identified as a protein affecting associative learning and/or memory [33], and was demonstrated to play an important role in regulating Ca^2+^-dependent synaptic plasticity [34,35]; whereas mouse Orc3 is required for dendritic growth [36]. Whether these Orc3-mediated functions are causative to schizophrenic symptoms need further evaluation. Second, a point mutation in *ORC4* gene is correlated with B-cell lymphoproliferative disorders, though the functional relevance needs to be elucidated [74]. Third, the *ORC5* gene has been linked to adult AML (acute myeloid leukaemia) and MDS (myelodysplastic syndrome), since its chromosomal location is within a region that is frequently deleted in myeloid malignancy patients. However, sequencing analyses of the remaining *ORC5* allele in AML or MDS patients with chromosomal deletions did not detect any mutations [75]. Fourth, ORCA/LRWD1 is highly expressed in testis [76,77], and *ORCA/LRWD1* gene may be a genetic risk to the sertoli cell-only syndrome [78]. These reports clearly suggest that individual mutations in ORC subunits and related factors are involved, directly or indirectly, in many human diseases. Further functional demonstrations on these correlations will be highly desired.

## EPSTEIN–BARR VIRUS (EBV): TAKING ADVANTAGE OF HOST ORC

In 1964, a herpesvirus-like particle was discovered in a cell line derived from a Burkitt's lymphoma biopsy by Epstein et al. in Barr group, and was therefore named EBV [79]. EBV is transmitted among people via saliva; and strikingly, more than 90% of the world population is infected by EBV [80]. As one of the most common viruses, most initial infections take place in childhood with no severe symptoms; and once infected, EBV can stay at its latent state infinitely in the host [81]. In the case that EBV becomes active, especially in people with immunodeficiency, it can cause a number of diseases, including epithelial malignancies, mesenchymal malignancies, lymphomas and lymphoproliferative disorders [80].

EBV contains a double-stranded DNA and replicates as an episome in latently infected cells. The duplication process requires the EBV-encoded protein EBNA1 (Epstein–Barr nuclear antigen 1) binding to its origin of viral replication oriP [82]. Interestingly, human cells with a hypomorphic mutation in *ORC2* (*ORC2* Δ/− cells) do not support the EBNA1-dependent replication of episomes from oriP, but this replication is restored upon expression of wild-type Orc2 [83]. Immunoprecipitations demonstrate Orc2 associates with EBNA1, whereas ChIPs (chromatin immunoprecipitations) reveal Orc2 binds to oriP [83,84], indicating that EBV utilizes EBNA1 to recruit host ORC to its origin in order to replicate its DNA. Moreover, ectopically expressing the replication licensing factor Geminin in human HCT116 cells (with the plasmid containing oriP and EBNA1) inhibits replication from oriP, and this can be rescued by co-expressing of Cdt1 [83]. Other studies reveal that Orc1 and MCM exhibit cell-cycle-regulated associations with oriP: they bind to oriP in G_1_ phase and dissociate from it during S phase, while other ORC subunits remain constantly bound [84,85]. Taken together, these data suggest that besides ORC, EBV hijacks the entire host pre-RC to carry out the EBNA1 and oriP-dependent replication, which is similarly restricted by the host replication licensing system.

Subsequent homology analyses of oriP and EBNA1 provide more details on how this virus uses ORC and associated factors to duplicate its genome. At the binding sequence level, studies on the oriP reveal two signature elements: the DS (dyad symmetry) and the FR (family of repeats), with the DS being the dominant but dispensable origin [86–89]. The minimal replicator of oriP has two EBNA-1 binding sites flanked by 14-bp inverted repeats; and a 9-bp core sequence (5′-TTAGGGTTA-3′) within the 14-bp repeat highly resembles the telomeric DNA sequence [90]. In fact, the indication that telomeric proteins are involved in ORC-dependent EBV replication has been confirmed by the discovery of the direct contribution from TRF2 (telomere repeat factor 2), PARP [poly(ADP-ribose) polymerase] and Chk2 [91–95]. At the protein level, investigations on the EBNA1 N-terminal domain identify two linking regions, LR1 and LR2 that interact with ORC, and the sequence analyses of LR1/2 regions show that they both have RG-rich motifs (arginine- and glycine-rich motifs) [96]. Deletion of LR1/2 from EBNA1 or substitution of arginines or glycines to alanines in LR2 region abolishes ORC association, indicating that the RG-rich motif is essential for ORC–EBNA1 interaction [96]. In addition, both EBNA1 and the RG-rich motif exhibit RNA binding abilities [97–99], and RNaseA but not DNaseI disrupts ORC–EBNA1 interaction [96], indicating that the EBNA1 recruits ORC in an RNA-dependent manner. Finally, the identity of this RNA is resolved as the G-quadruplex RNA [96,100].

In summary, EBV is an important example where the herpesvirus utilizes its own EBNA1 to recruit host ORC and replication initiation machinery, in coordination with auxiliary factors TRF2, PARP and Chk2, to duplicate its genome. With its defined origin sequence (oriP) and replication factors, it has become a powerful tool to study the initiation process of eukaryotic DNA replication [101–105]. As the cellular mechanisms unfold, one can develop effective targeted therapy to eradicate the EBV latency. For instance, based on the study of G-quadruplex RNA dependency in EBNA1–ORC interaction, it is reported that a G-quadruplex-interacting compound, BRACO-19, can abolish EBNA1 recruitment of ORC and inhibit EBNA1-dependent replication of oriP. Of significance, this abolishment preferentially blocks proliferation of EBV-positive cells compared with EBV-negative cell lines [100].

Another close member of the herpesviridae family is the KSHV (Kaposi's sarcoma-associated herpesvirus) that can infect tumour cells and stay latently in the host [106]. It contains a TR (terminal repeat) that serves as the replication origin (similar to oriP), and utilizes the LANA (latency-associated nuclear antigen; similar to EBNA1) to bind to TR to perform DNA replication [107–109]. Several studies have reported that ORC and MCM associate with TR [110–115], and Geminin has the similar inhibitory effect [113], suggesting that KSHV shares the comparable replication mechanism as that of the EBV episome: using the host's cellular replication machinery and staying with host's cell-cycle synchrony. Therefore eliminating the latent KSHV from the host highly relies on the depiction of detailed molecular mechanism and identification of additional targets. Recently, it is reported that a KSHV *cis*-acting DNA element within the LUR (long unique region) can initiate the replication of plasmids lacking a eukaryotic replication origin or LANA-binding sequence in a cellular pre-RC-dependent manner, indicating the existence of a novel functional origin independent of *trans*-acting viral proteins [115]. Additionally, histone acetyltransferase HBO1 (histone acetyltransferase binding to Orc1) and PARP1 were also discovered as TR-associating proteins [110,111]. Interestingly, LANA can be poly(ADP-ribosyl)ated by PARP1; compounds like HU (hydroxyurea), 3-AB (3-aminobenzamide) and NA (niacinamide) that alter PARP activity can affect KSHV viral copy number [110]. Since both EBV and KSHV are regulated by PARP, screening of drugs specifically targeting PARP activities would be a promising direction in eliminating viral latency in infected cells.

## AMERICAN TRYPANOSOMIASIS AND AFRICAN TRYPANOSOMIASIS: ARCHAEAL-LIKE ORC IN EUKARYOTES

American trypanosomiasis, also called Chagas’ disease, is a parasitic disease caused by the protozoan *Trypanosoma cruzi* [116]. *T. cruzi* is transmitted to humans by the insects, triatomines (also known as the ‘kissing bugs’) that feed on the blood from human faces. When triatomines bite on human skin, they pass *T. cruzi* parasites into their excrements that are left near the wounded skin, which allows parasites to enter and stay in the host's circulatory system [116]. Approximately ten million people, especially in the tropical area, are suffering from Chagas’ disease [116].

African trypanosomiasis, also called sleeping sickness, is another parasitic disease caused by *Trypanosoma brucei* [117]. The sleeping sickness can be further divided into acute and chronic types that are caused by two subspecies of *T. brucei*: *T. b. rhodesiense* and *T. b. gambiense*, respectively [117]. Infection by *T. b. rhodesiense* usually follows an animal-fly-human cycle, with the fast development (weeks) into host's CNS (central nervous system) [118]. Infection by *T. b. gambiense* instead follows a human–fly–human cycle, with a long duration (months or years) of infection, and it is this chronic sleeping sickness that accounts for approximately 95% of the total African trypanosomiasis cases [118].

Notably, both American and African trypanosomiasis can also be infected via other transmission manners, including blood transfusions, vertical transmission and accidental infections [118]. Currently, benznidazole and nifurtimox are the two common medicines used to treat American trypanosomiasis, whereas pentamidine, suramin, melarsoprol and eflornithine are the four common medicines used to treat African trypanosomiasis. However, they all face the issues of side effect, high toxicity, ineffective in chronic phase and drug resistance [116]. Therefore the search for new alternate becomes a necessity. Recent progress on the investigation of *T. cruzi* and *T. brucei* ORCs shed light on identifying novel drug targets.

Although trypanosomatids belong to eukaryotes, sequence alignment and structural analyses of *T. cruzi* and *T. brucei* protein database reveal that they both have protein sequences closer to archaeal Orc1/Cdc6 instead of the eukaryotic ORC, and are hence named TcOrc1/Cdc6 and TbOrc1/Cdc6 [119]. Orc1/Cdc6 is expressed in the nuclei of trypanosomes and is associated with chromatin throughout the cell cycle. Using thermosensitive yeast mutants, it is demonstrated that both TcOrc1/Cdc6 and TbOrc1/Cdc6 can complement the *cdc6* mutant, but not the *orc1* mutant [119]. Further, RNAi (RNA interference)-mediated depletion of TbOrc1/Cdc6 results in enucleated cells. Flow cytometry analyses on those cells reveal a sub-G_0_/G_1_ population (cells with no nucleus but only the kinetoplast) as well as a decreased population of G_2_/M cells, all indicating a hampered DNA replication [119]. Taken together, these data confirm that TcOrc1/Cdc6 and TbOrc1/Cdc6 are the components responsible for the initiation of replication in trypanosomes.

**Figure 2:**
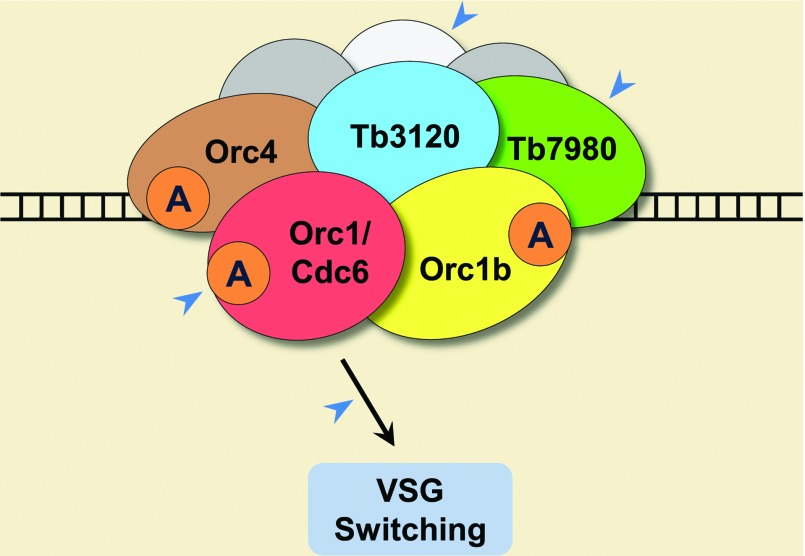
Replication initiation machinery in *Trypanosoma brucei* Besides the well-characterized TbOrc1/Cdc6, novel components like the Orc4 orthologue, Orc1-like protein Orc1b and new factors Tb7980 and Tb3120 have been described, although the underlying molecular mechanism of how they function in replication initiation remains to be elucidated. It is also possible that other ORC homologues or unknown factors exist (depicted in grey colour) to facilitate the replication. Many of these features could be potentially exploited as therapeutic targets (blue arrowheads), including: (i) the archaeal-like ATPase activities in TbOrc1/Cdc6, TbOrc1b and TbOrc4 (illustrated as ‘A’ in the circle); (ii) novel factors Tb7980 and Tb3120 that lack conserved AAA+ ATPases motifs; (iii) yet-to-identify factors facilitating TbORC functions; and (iv) the role of TbOrc1/Cdc6 in controlling VSG switching.

In a closer look at the protein sequences of TcOrc1/Cdc6 and TbOrc1/Cdc6, they both contain the signature sequences of Walker A and Walker B motifs as well as sensor I and II regions of the AAA+ family [119]. Recombinant TcOrc1/Cdc6 and TbOrc1/Cdc6 both exhibit ATP binding/hydrolysis activities, corroborating the *in silico* prediction. Moreover, in the presence of salmon sperm DNA, Orc1/Cdc6 displays enhanced ATPase activity [119], indicating that trypanosome Orc1/Cdc6 ATPase might be responsible for defining origin-binding specificity, similar to yeast Cdc6 [120]. A number of drugs targeting the bacterial type II topoisomerases DNA gyrase and topoisomerase IV are being used, based on the efficacy mainly as ATPase inhibitors [121]. Since *T. cruzi* and *T. brucei* use archaeal-like Orc1/Cdc6 as the initiation factor, screening of specific inhibitors that only target TcOrc1/Cdc6 and TbOrc1/Cdc6 ATPase activities but not that of human pre-RC would greatly benefit the treatment of American and African trypanosomiasis (Figure 2).

Recently, more insights into the replication initiation have been gained from the studies in *T. brucei*, rendering it an effective new model organism and providing more opportunities for the development of drug candidates. First, the identification and characterization of the CMG complex (Cdc45–MCM2–7–GINS) in *T. brucei* have been performed, and its requirement for DNA replication has also been demonstrated [122,123]. Secondly, another Orc1-like protein (Orc1b), bigger than Orc1/Cdc6 but smaller than the yeast Orc1 homologue, was identified. Similar to Orc1/Cdc6, Orc1b interacts with MCM proteins, indicating that *T. brucei* has two forms of Orc1-like proteins, Orc1/Cdc6 and Orc1b that both could potentially recruit MCM to form the pre-RC [122]. Thirdly, in search of Orc1/Cdc6-interacting factors, an Orc4 orthologue and two novel factors (Tb7980 and Tb3120) were identified. RNAi-mediated knockdown of these factors demonstrate that they are essential for the growth of *T. brucei* cells [123]. Since most of these newly identified factors are not conserved in mammalian cells, they could serve as potential drug targets of *T. brucei*-mediated African trypanosomiasis (Figure 2).

Interestingly, direct evidence of TbOrc1/Cdc6's function in DNA replication and telomere-linked VSG (variant surface glycoprotein) silencing and switching has been reported [124]. Each trypanosome can only express one type of VSG, and VSG is the only antigen that can be targeted by the host; consequently, trypanosomes try to escape from the host immune response by changing the expression to another VSG [125]. Therefore in addition to *T. brucei*'s own replication machinery, targeting the role of TbOrc1/Cdc6 in controlling VSG switching provides an alternative approach: restricting *T. brucei*'s VSG expression/switching and providing constant target for the host's immune system to eliminate *T. brucei* (Figure 2).

Based on the sequence homology, other protozoa like *Giardia lamblia* (one major cause of diarrhoeal diseases [126]) and *Leishmania major* (the pathogen for cutaneous leishmaniasis [127]) also have conserved Orc1/Cdc6 and Orc4 [123]. Therefore our knowledge gained from *T. cruzi* and *T. brucei* could also be applied to the improvement of diagnoses and/or treatments of these diseases.

## CONCLUSIONS AND PERSPECTIVES

The human body is constantly being attacked by internal mutations as well as external pathogens. ORC, as one most important complex in all eukaryotes, is inevitably involved in these processes and has been linked to both aspects in various diseases.

For those ORC-related genetic diseases, advancing biotechnology seems a must. On the one hand, family-based genome/exome sequencing makes the identification of numerous SNP a highly effective approach [128]. As demonstrated from the study on Orc1 and Orc4 mutations in MGS, follow-up functional analyses based on the SNP can now be performed and the mechanistic links are beginning to be appreciated. On the other hand, with many mutations diagnosed and characterized in different ORC-associated diseases, the urge for replacing the mutated genes with functional ones through gene therapy warrants the therapeutic focus.

For those pathogen-mediated diseases, one should take advantage of the unique characteristics of pathogens. As the γ herpesvirinae subfamily members of the herpesvirus, EBV and KSHV share common mechanism for latent origin replication: utilizing host's cellular replication machinery and being subjected to licensing system regulation [115]. Therefore targeting EBV/KSHV-specific factors (like compounds targeting G-quadruplex RNA and PARP activity) illuminates an alternative route to eliminating their latency. *T. cruzi* and *T. brucei* use archaeal-like Orc1/Cdc6 in the host system, so targeting archaeal Orc1/Cdc6 ATPase activity but not that of human pre-replication complex may be a feasible angle.
